# Layer-number-controlled Fe-tea polyphenol metal-phenolic network-coated PLCL/gelatin electrospun membranes for active preservation of yellow sweet cherries

**DOI:** 10.1016/j.fochx.2026.104602

**Published:** 2026-10-21

**Authors:** Jiaqi Liu, Tingzi Yan, Qiongyu Wang, Jiaxuan Bao, Yuchan Li, Jigang Piao, Hui Chai, Guoying Zhou

**Affiliations:** a College of Life Science, Zhejiang Chinese Medical University, Hangzhou 310053, China; b College of Material, Chemistry and Chemical Engineering, Hangzhou Normal University, Hangzhou 311121, China; c College of Pharmacy, Zhejiang Chinese Medical University, Hangzhou 310053, China

**Keywords:** Electrospinning, PLCL/gelatin membrane, Metal-phenolic networks, Tea polyphenol, Active packaging

## Abstract

To address postharvest fruit spoilage from microbial contamination and oxidation, biodegradable PLCL/gelatin electrospun membranes were functionalized with layer-number-controlled Fe-tea polyphenol (Fe-TP) networks via layer-by-layer assembly. Fe-TP deposition increased fiber diameter, improved hydrophilicity, and enhanced mechanical strength while maintaining flexibility. Increasing the number of assembly cycles enhanced TP release and antioxidant activity, delayed degradation, and improved antibacterial activity against Gram-positive and Gram-negative bacteria. Cytocompatibility and hemolysis assays indicated favorable biosafety. SEM and leakage assays suggested membrane disruption and intracellular leakage as possible antibacterial mechanisms. In yellow sweet cherry preservation, the Fe-TP effectively delayed decay. Although Fe-TP/PLCL-Gel 8 exhibited stronger antibacterial and hydrophilic performance, the Fe-TP/PLCL-Gel 4 achieved the best overall balance of mechanical properties, release, safety, and preservation efficacy, with a day-7 decay rate of only 1.85% ± 3.21%, versus 50.00% ± 5.56% for PE control. This layer-optimized multifunctional electrospun membrane offers a highly effective, promising material for fruit preservation.

## Highlights

• Layer-controlled Fe-TP coating endows PLCL/Gel fibers with favorable physical properties.
• Fe-TP coating confers potent antioxidant and antibacterial activities and excellent biocompatibility.
• Fe-TP/PLCL-Gel 4 exhibits optimal preservation performance for yellow sweet cherries.

## Graphical abstract

Schematic diagram of experimental process and mechanism.

**Figure:**
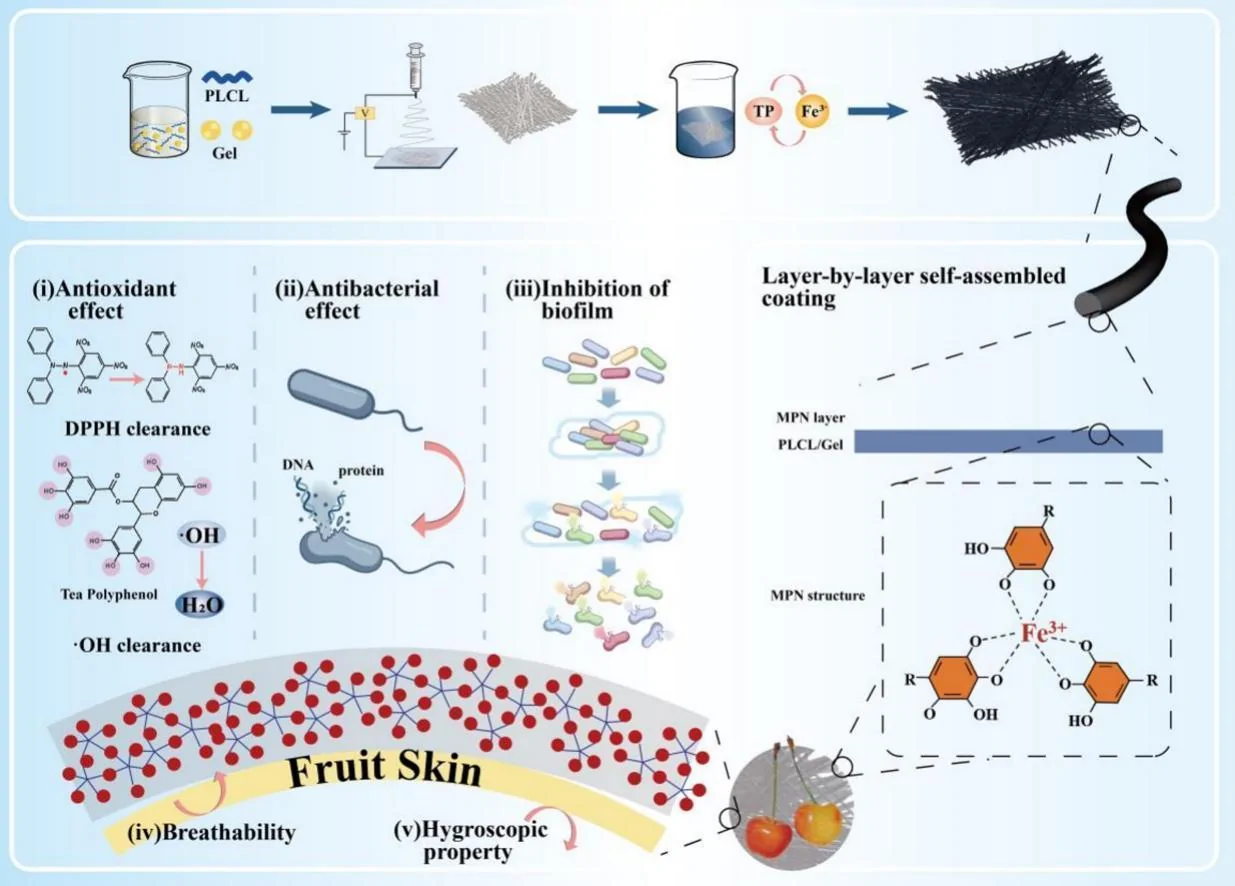

## 1 Introduction

Postharvest losses of fresh fruits and vegetables remain a major challenge for food supply chains and resource utilization. During storage and transportation, fresh produce is vulnerable to microbial contamination, oxidative browning, moisture migration, and abnormal respiratory metabolism, which together accelerate quality deterioration and shorten shelf life (Álvarez-Hernández et al., 2021). Sweet cherry (*Prunus avium* L.) is particularly perishable because of its thin skin, high transpiration rate, susceptibility to mechanical damage, and sensitivity to fungal infection. These characteristics often lead to water loss, softening, browning, and mold development after harvest, making sweet cherry a representative model for evaluating advanced preservation packaging systems (Yuan et al., 2023). Therefore, sustainable packaging materials that can provide both physical protection and active preservation functions are urgently needed.

Conventional packaging materials, especially petroleum-based plastics, mainly function as passive barriers but lack the ability to inhibit microbial growth, suppress oxidative deterioration, or regulate the storage microenvironment (Ma et al., 2022). Moreover, they are not inherently degradable, which leads to a severe environmental burden. Therefore, the development of biodegradable active food packaging has been strongly stimulated. In contrast to conventional passive materials, active food packaging integrates functional components capable of interacting with food products or the surrounding environment, for example by releasing antimicrobial or antioxidant agents or by regulating oxygen, ethylene, and moisture levels (Mahmud et al., 2022; Pawase et al., 2025; Syed et al., 2025). Among different active packaging strategies, metal-phenolic networks (MPNs) have attracted increasing attention because they can be formed rapidly under mild conditions through coordination interactions between metal ions and natural polyphenols (Li, Cao, et al., 2025). More importantly, their coating thickness, surface chemistry, wettability, and active-component loading can be tuned by adjusting the number of layer-by-layer assembly cycles (He et al., 2025; Lin et al., 2024; Wang et al., 2025).

Tea polyphenols (TP) are natural bioactive compounds with strong antioxidant and antimicrobial activities. They can scavenge reactive oxygen species, inhibit lipid oxidation, and damage bacterial cell membranes or interfere with intracellular metabolism, thereby showing potential for food preservation (Gao et al., 2025; Ma et al., 2024; Xu et al., 2025). However, direct incorporation of TP into packaging materials or coatings is often limited by their high water solubility, rapid migration, limited stability, and insufficient long-term utilization (Wang, Cao, et al., 2024). Coordination assembly with Fe^3+^ offers an effective strategy to improve the interfacial retention and controlled release of TP through the formation of Fe^3+^-TP MPNs (Cheng et al., 2026). In our previous work, a single-layer Fe^3+^-TP assembly was shown to improve sweet cherry preservation, but the influence of Fe^3+^-TP assembly cycles on coating structure, release behavior, functional activity, and preservation efficacy was not systematically investigated (Dou et al., 2026). This knowledge gap limits the rational design of layer-number-controlled active packaging systems.

Electrospun membranes provide a suitable platform for constructing such active packaging interfaces (Mohammadi et al., 2024). Owing to their high specific surface area, interconnected porous structure, flexibility, and tunable fiber morphology, electrospun membranes can offer abundant active sites for functional deposition and efficient contact with food surfaces (Min et al., 2022; Nanda et al., 2024; Sameen et al., 2022). Compared with dense films, fibrous membranes are more favorable for interfacial immobilization and active-component utilization. In particular, poly (l-lactide-*co*-caprolactone) (PLCL) can provide flexibility and biodegradability, while gelatin contributes hydrophilicity, biocompatibility, and functional groups for interfacial interactions. Therefore, PLCL/gelatin electrospun membranes are promising biodegradable substrates for Fe^3+^-TP MPN modification, enabling the integration of structural support, sustained release, antimicrobial activity, antioxidant activity, and fruit preservation functions (Xie et al., 2024).

This work aims to establish a layer-number-dependent structure-function-preservation relationship and to identify a balanced design strategy for biodegradable active packaging membranes. Herein, PLCL/gelatin electrospun membranes were fabricated and further functionalized with Fe-TP MPN coatings through a layer-by-layer assembly strategy. By varying the number of Fe-TP assembly cycles, the relationship between MPN layer number and membrane morphology, chemical structure, mechanical performance, wettability, TP release, degradation behavior, antioxidant activity, antibacterial and anti-biofilm performance, preliminary biocompatibility, and yellow sweet cherry preservation was systematically evaluated. We present a simple PLCL/gelatin membrane with a layer-by-layer stacked Fe–TP coating, enabling tunable loading and multifunctional preservation of sweet cherries, highlighting its great potential for fruit packaging (Fig. 1).

**Fig. 1:**
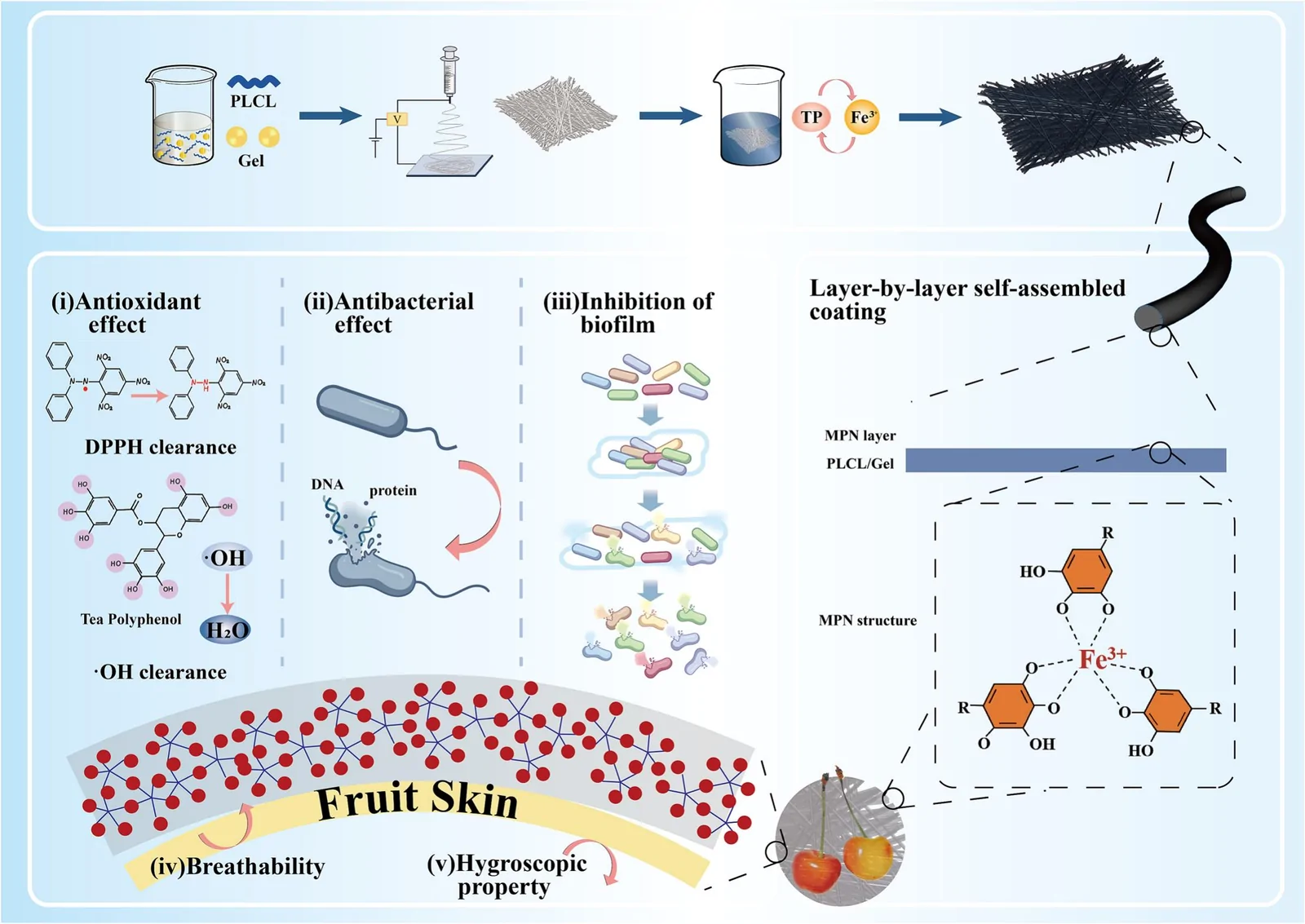
Schematic diagram of the formation of Fe-TP/PLCL-Gel electrospun membrane and its mechanism for fruit preservation of sweet cherries.

## 2 Materials and methods

### 2.1 Materials, cells, and strains

Hexafluoroisopropanol (HFIP), 1,1-diphenyl-2-picrylhydrazyl (DPPH), tea polyphenol (TP), and ferric chloride hexahydrate (FeCl₃·6H₂O) were purchased from Aladdin Biochemical Technology Co., Ltd. (Shanghai, China). Poly(l-lactide-*co*-ε-caprolactone) (PLCL, 50:50) was purchased from Jinan Daigang Biological Engineering Co., Ltd. (Jinan, China). Gelatin was purchased from Sigma-Aldrich (St. Louis, MO, USA). Dulbecco's modified Eagle's medium (DMEM), fetal bovine serum (FBS), horse serum (HS), The CCK-8 assay kit was purchased from Beijing Lanjie Technology Co., Ltd. (Beijing, China). Phosphate-buffered saline (PBS) powder and 0.1% crystal violet and hydroxyl radical scavenging assay kit were purchased from Beijing Solarbio Science & Technology Co., Ltd. (Beijing, China).

Mouse fibroblast cells (L929) were obtained from the China Center for Type Culture Collection (Wuhan, China). *Escherichia coli* (*E. coli*) ATCC 25922 and *Staphylococcus aureus* (*S. aureus*) ATCC 25923 were originally obtained from ATCC and maintained in the microbiology laboratory of Zhejiang Chinese Medical University. Mature sweet cherries were purchased from a local market and selected based on uniform appearance and weight. The fruits were used directly without washing or disinfection prior to the experiment.

### 2.2 Preparation of PLCL/Gel electrospun membranes

PLCL/gelatin membranes were prepared by conventional electrospinning. PLCL and gelatin were separately dissolved in hexafluoroisopropanol (HFIP) under magnetic stirring. The weight ratio of PLCL to gelatin was 4:1 (0.64 g PLCL in 10 mL HFIP and 0.16 g gelatin in 1.6 mL HFIP), giving a final polymer concentration of ∼6.9% (*w*/*v*). The gelatin solution was added dropwise to the PLCL solution under continuous stirring until homogeneous. Aluminum foil on the collector served as the receiving substrate. Electrospinning used a 20-gauge needle at a distance of 13 cm, with a positive voltage of 6.37 kV, a negative voltage of 3.58 kV, and a solution feed rate of 0.126 mm/min (volumetric flow rate ∼ 0.85 mL/h for a 5 mL syringe, inner diameter ∼ 12 mm). Under these conditions, each membrane was collected for ∼1.5 h and stored for Fe-TP modification and characterization.

### 2.3 Layer-by-layer assembly of Fe-TP coatings on PLCL/Gel electrospun membranes

Fe-TP coatings were deposited onto PLCL/Gelatin electrospun membranes via layer-by-layer self-assembly. Coated membranes were denoted as Fe-TP/PLCL-Gel x, where x is the number of assembly cycles (one cycle = one TP immersion + one Fe immersion).

FeCl₃·6H₂O and TP were dissolved in ultrapure water at 10 mg/mL and 40 mg/mL, respectively (Fe^3+^:TP mass ratio = 1:4). For each cycle, membranes were immersed in 20 mL TP solution for 15 min, rinsed with deionized water, blotted with filter paper, then immersed in 20 mL Fe^3+^ solution for 15 min, followed by rinsing and blotting. Repeating this procedure for different cycles yielded Fe-TP/PLCL-Gel 1, 4, and 8. After the final cycle, membranes were blotted and freeze-dried for characterization.

### 2.4 Characterization of Fe-TP/PLCL-Gel electrospun membranes

The morphology of the electrospun membranes (uncoated and Fe-TP-coated) was observed using scanning electron microscopy (SEM; SU8010, Hitachi, Japan). Samples were sputter-coated with gold before imaging. Images were taken from random regions, and fiber diameters were analyzed using ImageJ. Chemical structure was examined by Fourier transform infrared spectroscopy (FTIR; Nicolet iS50, Thermo Fisher, USA) to verify successful Fe-TP deposition. X-ray photoelectron spectroscopy (XPS; Nexsa G2, Thermo Fisher Scientific, USA) was further employed to analyze the surface chemical states of Fe on the fibrous membranes and to determine the Fe valence state distribution. The uncoated and Fe-TP-coated membranes were cut into 5 × 5 mm specimens, and five spots per membrane were randomly selected and measured, with three replicates per group. Surface wettability was evaluated by water contact angle measurements usinga droplet shape analyzer (DSA-100, KRÜSS, Germany). Briefly, a 3 μL water droplet was placed on the membrane surface, and the contact angle was recorded at ≥3 sites per sample. The mean and standard deviation were calculated from measurements performed in triplicate.

### 2.5 Mechanical properties, loading content, release kinetics, and soil burial degradation of Fe-TP/PLCL-Gel electrospun membranes

Mechanical properties were evaluated using a universal testing machine (5966, INSTRON, USA). Tensile specimens (type I) were prepared with a cutting mold (J03–0.3 T, Shanghai Shenkang, China). Tests were conducted at a maximum load of 500 N and a tensile speed of 10 mm/min. Tensile strength, elongation at break, elastic modulus, and maximum load were obtained from stress–strain curves. At least three specimens per group were tested.

The cross-sectional morphology of the membranes was examined by SEM to visualize the distribution and deposition of Fe-TP. Samples were sputter-coated with gold before imaging.

Loading content was assessed by gravimetric analysis, Folin–Ciocalteu assay, and ICP-OES. For gravimetric analysis, freeze-dried 2 × 3 cm specimens of comparable thickness were weighed before and after Fe-TP loading (*n* = 3), and the total loaded mass was calculated from the mass difference. TP loading was determined by a modified Folin–Ciocalteu assay (Raposo et al., 2024). EDTA was used to release TP by competing with polyphenols for Fe^3+^ chelation (Chen et al., 2024). Briefly, membrane samples (50 mg each, *n* = 3) were cut into small pieces and extracted three times with 30 mL of 50 mmol/L EDTA (pH 7.2), each under ice-bath sonication for 1 h followed by shaking for 12 h. The extracts were combined and filtered (0.22 μm). An aliquot of the combined extract was mixed with an equal volume of methanol (final 50%, *v*/v). The TP amount in a fourth extraction with 10 mL of EDTA was less than 5% of that in the combined first three extracts, indicating that additional extraction contributed negligibly to the total TP recovery. TP was quantified at 765 nm using matrix-matched calibration. Fe loading was measured by ICP-OES (Agilent 5800, Agilent Technologies, Malaysia) after acid digestion of 150 mg samples (*n* = 3) and expressed as mg Fe per mg membrane.

TP release was evaluated in phosphate-buffered saline (PBS, pH 7.4) at 37 °C and 180 rpm in the dark. Membrane samples with different coating cycles were immersed in 5 mL prewarmed PBS (37 °C). At predetermined time points (0.5, 1, 2, 4, 6, 24, 30, 48, and 72 h), 0.5 mL of release medium was collected and replaced with fresh PBS. TP concentration was determined by UV–vis spectrophotometry at 275 nm using a standard calibration curve. Cumulative release was expressed as TP concentration.

Degradation was evaluated using a soil burial assay. Samples were buried in neutral soil at 25 °C and 60% relative humidity. Membranes were recovered on days 10, 20, and 30 (three samples per time point). After washing and drying, residual mass was recorded, and degradation performance was assessed by mass loss. Degradation rate was calculated as follows:

(1) $$\text{Degradation rate}\;\left(\%\right)=\frac{\left(W0-\mathrm{Wt}\right)}{W0}\times 100\%$$

where W_0_ represents the initial mass of the membranes, and W_t_ represents the mass of the membranes at each degradation time point.

### 2.6 Antioxidant activity of Fe-TP/PLCL-Gel electrospun membranes

Antioxidant activity was evaluated by DPPH and hydroxyl radical scavenging assays. For the DPPH assay, membrane samples (6 mg) were extracted with 3 mL of methanol in the dark for 6 h. Ultrapure water (0.5 mL) was used as the control and was treated with 3 mL of methanol under the same conditions. Then, 50 μL of extract was mixed with 150 μL of 0.2 mM DPPH methanolic solution in a 96-well plate and incubated for 30 min in the dark. Absorbance was measured at 517 nm. A blank (without DPPH) corrected for intrinsic sample color. DPPH scavenging activity was calculated as follows:

(2) $$\text{DPPH scavenging efficiency}\;\left(\%\right)=\frac{A_{c}‐\left(A_{s}-A_{b}\right)}{A_{c}}\times 100\%$$

where A_c_ is the absorbance of the DPPH solution mixed with solvent, A_s_ is the absorbance of the sample extract mixed with DPPH solution, and A_b_ is the absorbance of the corresponding sample extract without DPPH.

Hydroxyl radical scavenging activity was measured using a commercial kit (BC1320, Solarbio, China). For the sample group, 0.75 mL working solution, 0.5 mL membrane extract, 0.15 mL reagent IV, and 0.45 mL water were mixed. The blank group contained 0.75 mL working solution and 0.75 mL water. The control group contained 0.75 mL working solution, 0.15 mL reagent IV, and 0.60 mL water. After incubation at 37 °C for 60 min, absorbance was recorded at 536 nm. Scavenging activity was calculated as follows:

(3) $$\mathrm{OH}\;\text{scavenging activity}\;\left(\%\right)=\frac{A_{S}-A_{C}}{A_{B}-A_{C}}\times 100\%$$

where A_c_, A_s_ and A_b_ represent the absorbance values of the sample, control, and blank groups, respectively. All assays were performed in triplicate.

### 2.7 Antibacterial activity and biofilm inhibition of Fe-TP/PLCL-Gel electrospun membranes

Antibacterial activity against *Escherichia coli* ATCC 25922 and *Staphylococcus aureus* ATCC 25923 was evaluated by co-incubation followed by plate counting. Bacteria were cultured to logarithmic phase (OD₆₀₀ = 0.6–0.7) and adjusted to 10^6^ CFU/mL. Membrane samples (20 mg) were immersed in 4 mL of bacterial suspension and co-incubated at 37 °C for 4 h with shaking. The suspensions were serially diluted to 10^−5^, and 100 μL was spread on LB agar plates (10 cm). Colonies were counted after 24 h at 37 °C. Antibacterial efficiency (%) was calculated as follows:

(4) $$\text{Antimicrobial efficiency}\;\left(\%\right)=\frac{C_{0}-C_{t}}{C_{0}}\times 100\%$$

here, C_t_ and C_0_ represents the colony counts of the experimental and control groups, respectively.

Biofilm inhibition was assessed via crystal violet staining. Bacteria were cultured to logarithmic phase (OD₆₀₀ = 0.6–0.7), centrifuged, resuspended to OD₆₀₀ = 1, and diluted 1:100 in LB. Aliquots (500 μL) were seeded into 24-well plates and incubated for 48 h to form biofilms. The medium was then replaced with 500 μL of membrane extract, prepared by immersing 10 mg of membrane in 2 mL LB for 48 h, and biofilms were incubated for another 48 h. After treatment, 500 μL of 0.1% crystal violet was added per well for 15 min. The stained biofilms were dissolved in 500 μL of 95% ethanol for 5 min, and absorbance was measured at 570 nm. The biofilm inhibition rate (%) was calculated as follows:

(5) $$\text{Biofilm inhibition rate}\;\left(\%\right)=\frac{A_{0}-A_{t}}{A_{0}}\times 100\%$$

where A_0_ and A_t_ denote the absorbance values of the control and experimental groups, respectively.

Bacterial viability was visualized by DMAO/PI live/dead staining. Bacterial suspensions (10^8^ cells/mL) were mixed with the staining working solution (1 μL per 100 μL suspension), incubated at 37 °C in the dark for 20 min, and observed under a fluorescence microscope.

To investigate the antibacterial mechanism, SEM and intracellular leakage assays were performed. For SEM, membrane samples (20 mg) were immersed in 1.2 mL LB broth and 1.5 mL bacterial suspension, incubated at 37 °C for 2 h, and fixed with 2.5% glutaraldehyde at 4 °C overnight. The samples were dehydrated through graded ethanol (30–100%), sputter-coated with gold, and observed by SEM.

For leakage analysis, bacteria (10^6^ CFU/mL, 2 mL) were centrifuged (8000 rpm, 10 min), washed three times with PBS, and resuspended in PBS to OD₆₀₀ ≈ 0.1. After incubation with membranes with shaking, supernatants were collected at 1 and 5 h. Nucleic acid release was measured at 260 nm at 1 and 5 h, and protein release was measured at 5 h by BCA assay. PBS and Triton X-100 served as negative and positive controls, respectively. Relative leakage (%) was calculated as follows:

(6) $$\text{Relative leakage}\left(\%\right)=\frac{\left(\mathrm{Ae}-\mathrm{Ap}\right)}{\left(\mathrm{Ax}-\mathrm{Ap}\right)}\times 100\%$$

where A_e_ denotes the absorbance of the experimental group, A_x_ denotes the absorbance of the Triton X-100 positive control, and A_p_ denotes the absorbance of the PBS negative control.

### 2.8 Cytocompatibility and hemocompatibility evaluation of Fe-TP/PLCL-Gel electrospun membranes

Cytocompatibility was evaluated using L929 mouse fibroblasts via the CCK-8 assay. Cells were cultured under standard conditions and exposed to membrane extracts or direct contact. After incubation, CCK-8 solution was added, and absorbance was measured to determine cell viability. Untreated cells served as the control, and cell-free medium as the blank. Viability was expressed as a percentage relative to the control and calculated as follows:

(7) $$\text{Cell viability}\;\left(\%\right)=\frac{\left(\text{ODtest}\;‐\;\text{ODblank}\right)}{\text{ODcontrol}}\times 100\%$$

where O_test_, O_blank_, and O_control_ were the absorbance of the test, blank, and control, respectively.

Blood compatibility was evaluated using a hemolysis assay. After incubation at 37 °C for 1 h, the samples were photographed, then centrifuged at 2000 rpm for 5 min. Supernatant (100 μL) from each sample was transferred to a 96-well plate, and absorbance was measured at 540 nm. PBS and 0.1% Triton X-100 served as negative and positive controls, respectively. The hemolysis rate was calculated as follows:

(8) $$\text{Hemolysis rate}\;\left(\%\right)=\frac{\left(\mathrm{Ae}\;-\;\mathrm{Ap}\right)}{\left(\mathrm{Ax}\;-\;\mathrm{Ap}\right)}\times 100\%$$

where Ae denotes the absorbance of the experimental group, Ax denotes the absorbance of the Triton X-100 positive control, and Ap denotes the absorbance of the PBS negative control.

### 2.9 Preservation performance of Fe-TP/PLCL-Gel electrospun membranes on yellow cherries

Uniform cherries were randomly placed in identical ASONE weighing dishes. PE cling film and each composite preservative film were used to cover the containers, with the film in direct contact with the cherry surface. Storage lasted 7 days. Mass retention rate (%) was calculated as follows:

(9) $$\text{Mass retention rate}\left(\%\right)=\frac{M_{2}}{M_{1}}\times 100\%$$

where M₁ represents the initial mass of the fruit (g), and M₂ represents the mass of the fruit after storage (g).

Fruit decay was evaluated according to the method described by Gao et al.(Gao et al., 2022) as follows: Grade 0, no visible decay; Grade 1, decay area less than 25% of the fruit surface; Grade 2, decay area between 25% and 50% of the fruit surface; and Grade 3, decay area greater than 50% of the fruit surface. The fruit decay rate was calculated as follows:

(10) $$\text{Decay rate}\left(\%\right)=\frac{\sum \left(C_{t}\times D_{t}\right)}{C_{0}\times D_{\max}}\times 100\%$$

where C_t_ represents the number of rotten fruits at each grade, D_t_ represents the grade value corresponding to the fruit, C_0_ represents the total number of fruits, and D_max_ represents the maximum grade value.

### 2.10 Statistical analysis

All data in this experiment were presented as mean ± standard deviation (SD). Statistical analyses were performed using one-way ANOVA for comparisons among multiple groups and two-way ANOVA where two factors were involved, followed by Tukey's post hoc test. Differences were considered statistically significant at *p* < 0.05, with significance levels indicated as *p < 0.05, **p < 0.01, ***p < 0.001, and ****p < 0.0001. Data analysis and graphing were performed using GraphPad Prism 10 and Origin 2024 software.

The normalization method and weighting strategy for the radar plot (Fig. 7 E) are described as follows. To enable visual comparison of indicators with different units and scales, all indicators in the radar plot were min–max normalized to a 0–100 scale across the compared groups (PLCL-Gel, Fe-TP/PLCL-Gel 1, 4, and 8). For benefit-type indicators (higher values preferable), including elastic modulus, TP release at 72 h, degradation stability at 10 d, DPPH and ·OH scavenging rates, cytocompatibility at 5 d, antibacterial activity, and weight remaining rate at 7 d, the normalized value was calculated as:

(11) $$\text{Normalized value}\;\left(\%\right)=\frac{X-X_{\min}}{X_{\max}-X_{\min}}\times 100\%$$

For cost-type indicators (lower values are preferable), specifically water contact angle and decay rate at 7 d, the normalized value was calculated as:

(12) $$\text{Normalized value}\;\left(\%\right)=\frac{X_{\max}-X}{X_{\max}-X_{\min}}\times 100\%$$

All axes were displayed with equal visual weight. No weighted composite score or inferential statistical test was derived from the radar area. The radar plot is intended as a descriptive visualization of multi-indicator performance. This normalization was used solely for visualization and did not alter the statistical analysis of individual indicators.

## 3 Results

### 3.1 Morphological and structural characterization of the electrospun membranes

The surface morphologies of the electrospun nanofibrous membranes treated with different Fe-TP assembly cycles were observed by scanning electron microscopy (SEM). As shown in Fig. 2 A, the fiber surface gradually transformed from relatively smooth to rough with increasing Fe-TP concentration. In addition, the fiber diameter and its distribution were statistically analyzed using ImageJ software (Fig. 2 B). The average diameters of the PLCL-Gel, Fe-TP/PLCL-Gel 1, Fe-TP/PLCL-Gel 4, and Fe-TP/PLCL-Gel 8 membranes were 886.08 ± 247.59 nm, 852.95 ± 217.92 nm, 1411.17 ± 237.38 nm, and 1469.76 ± 342.05 nm, respectively. These results indicate that the average fiber diameter increased significantly with increasing Fe-TP assembly cycles. Notably, the Fe-TP/PLCL-Gel 4 exhibited a relatively narrow size distribution while maintaining a significantly increased diameter, indicating a more uniform surface coating and better structural stability.

**Fig. 2:**
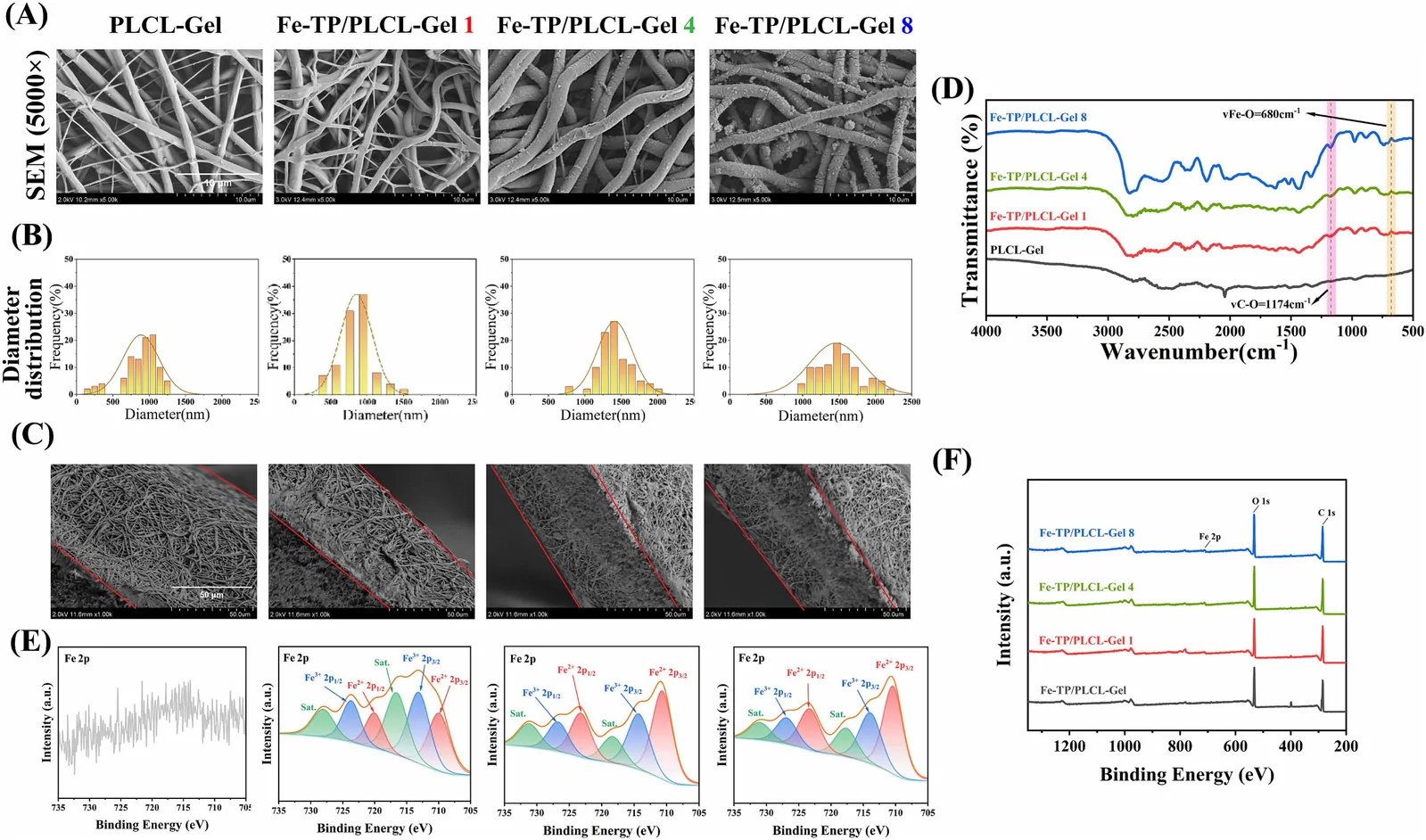
Morphological and surface chemical characterization of Fe-TP/PLCL-Gel electrospun membranes. (A) Surface SEM images of membranes with different assembly cycles (scale bar = 10 μm); (B) Fiber diameter distributions; (C) Cross-sectional SEM images of the electrospun membranes, the two red lines indicate the upper and lower boundaries of the fibrous membrane cross-sections (scale bar = 50 μm); (D) FTIR spectra; (E) XPS spectra in Fe 2p regions; (F) XPS survey spectra. (For interpretation of the references to color in this figure legend, the reader is referred to the web version of this article.)

The cross-sectional morphologies of the membranes were further examined by SEM (Fig. 2 C). The cross-sectional images showed that the Fe-TP coating was deposited primarily from the surface inward. As the number of assembly cycles increased, the loaded layer became denser without an obvious change in the overall membrane width, suggesting tighter complexation within the Fe-TP network at higher assembly cycles. These observations indicate that the Fe-TP coating was successfully deposited on the fiber surface while preserving the overall fibrous structure.

### 3.2 Surface chemistry and loading content of Fe-TP/PLCL-Gel nanofibrous membranes

The Fourier transform infrared (FTIR) spectra of the nanofibrous membranes are shown in Fig. 2 D. The weak absorption peak at 680 cm^−1^ can be assigned to the Fe—O bond(Tavakol et al., 2020), indicating coordination between Fe and TP. The prominent peak at 1174 cm^−1^ corresponds to the C—O stretching vibration of phenolic compounds in the Fe-TP complex, and its intensity increased with increasing TP loading cycles, further confirming the successful incorporation of TP(Zhao et al., 2026). Taken together, the presence of characteristic peaks associated with TP and the Fe-TP metal–polyphenol coordination complex demonstrates the successful fabrication of the Fe-TP/PLCL-Gel electrospun membranes.

XPS was applied to probe the surface chemistry of the membranes (Fig. 2 E). The survey spectra showed C, N, O, and Fe in Fe-TP/PLCL-Gel 1, 4, and 8, whereas the bare PLCL-Gel membrane showed no detectable Fe 2p signal, confirming that Fe was introduced only after loading. In the Fe 2p region, all loaded samples exhibited two peaks at ca. 710 and 724 eV, corresponding to Fe 2p₃/₂ and Fe 2p₁/₂ (Matías-Reyes et al., 2023). Deconvolution revealed both Fe^2+^ and Fe^3+^ species. The Fe^2+^ fraction increased from 44.95% for Fe-TP/PLCL-Gel 1 to 59.22% and 60.79% for Fe-TP/PLCL-Gel 4 and 8, respectively, while the Fe^3+^ fraction decreased correspondingly. These results confirm successful Fe loading and a mixed Fe^2+^/Fe^3+^ surface, with similar Fe 2p spectra for Fe-TP/PLCL-Gel 4 and 8. The corresponding full survey spectra are provided in Fig. 2F.

The Fe loading content was quantified by inductively coupled plasma optical emission spectrometry (ICP-OES) in Fig. 3 A. The Fe loading increased progressively with the number of assembly cycles, reaching 3.233 ± 0.1522, 4.790 ± 4.790, and 7.262 ± 0.2099 μg mg^−1^ for Fe-TP/PLCL-Gel 1, 4, and 8, respectively. The TP loading content was determined by the Folin–Ciocalteu method (Fig. 3 B). The TP loading also increased markedly with assembly cycles, with values of 2.920 ± 0.6789, 15.08 ± 2.684, and 28.94 ± 5.833 mg mg^−1^ for Fe-TP/PLCL-Gel 1, 4, and 8, respectively. The mass gain after Fe-TP loading followed the same trend (Fig. 3 C), increasing from 3.233 ± 0.1155 mg mg^−1^ for Fe-TP/PLCL-Gel 1 to 13.67 ± 3.365 mg mg^−1^ for Fe-TP/PLCL-Gel 4 and 33.00 ± 4.180 mg mg^−1^ for Fe-TP/PLCL-Gel 8. These results confirm that the Fe-TP coating was successfully deposited on the PLCL-Gel electrospun membranes, and the loading content could be tuned by controlling the number of assembly cycles.

**Fig. 3:**
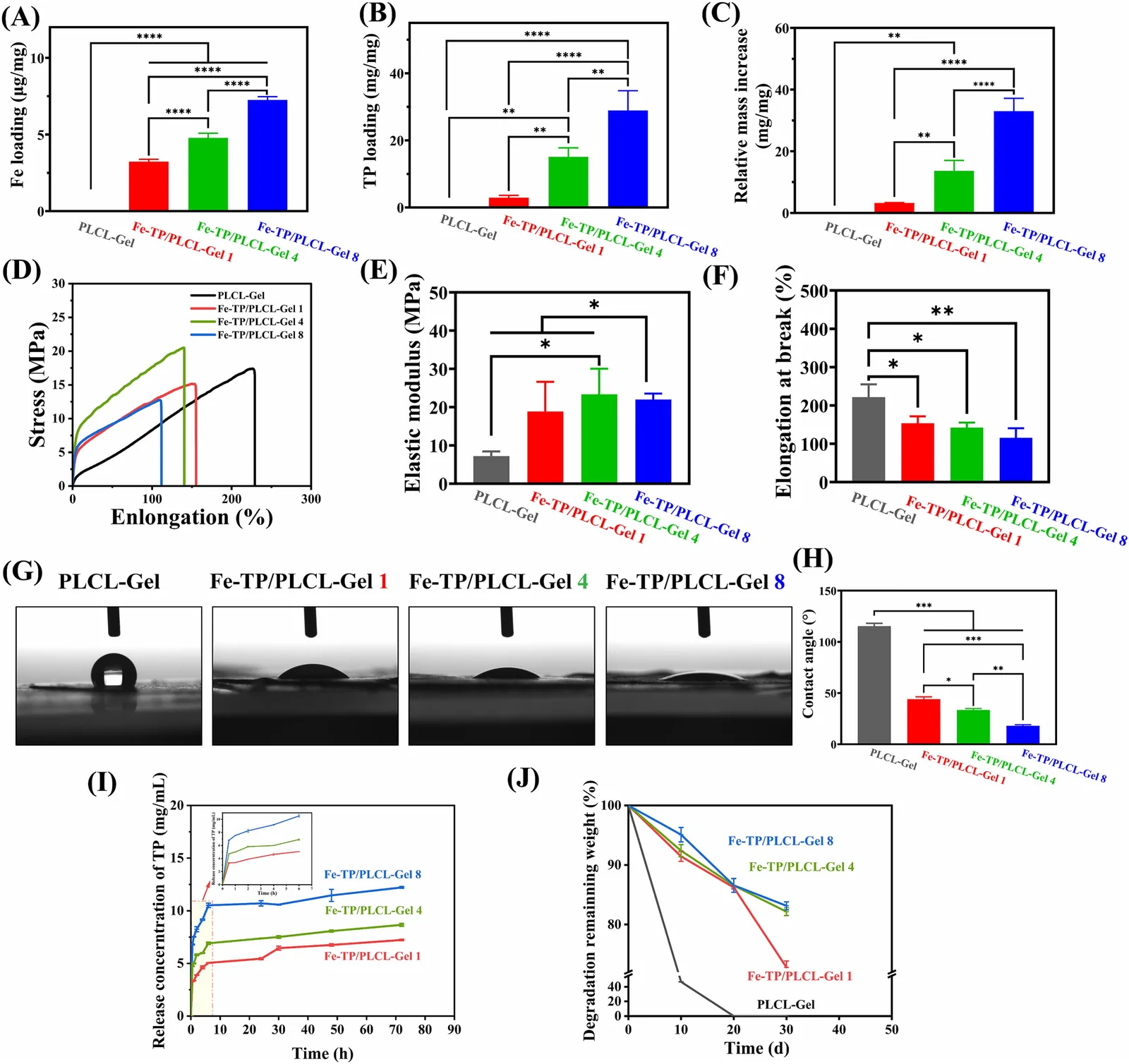
Loading content and physicochemical properties of Fe-TP/PLCL-Gel electrospun membranes. (A) Fe loading; (B) TP loading; (C) Mass gain after Fe-TP loading; (D) Stress–strain curves; (E) Elastic modulus; (F) Elongation at break; (G and H) Water contact angle images and values; (I) TP release profiles; (J) Soil-burial degradation profiles. Data are mean ± SD (*n* = 3). *p < 0.05, **p < 0.01, ***p < 0.001, ****p < 0.0001.

### 3.3 Mechanical properties and water wettability of Fe-TP/PLCL-Gel nanofibrous membranes

Uniaxial tensile testing was performed to evaluate the influence of Fe-TP assembly cycles on the mechanical behavior of the PLCL-Gel nanofibrous membranes(Dolgin et al., 2023). As shown in Fig. 3D–F, the pristine PLCL-Gel membrane exhibited the highest elongation at break (221.6 ± 33.9%), while all Fe-TP-modified membranes remained flexible with elongation at break above 100% (154.0 ± 17.9%, 142.2 ± 13.21%, and 115.8 ± 24.56% for Fe-TP/PLCL-Gel 1, 4, and 8, respectively). Among the modified membranes, Fe-TP/PLCL-Gel 4 exhibited the highest tensile stress and elastic modulus. The elastic modulus increased from 18.87 MPa for Fe-TP/PLCL-Gel 1 to 23.33 MPa for Fe-TP/PLCL-Gel 4, and then slightly decreased to 22.02 MPa for Fe-TP/PLCL-Gel 8, while remaining higher than that of Fe-TP/PLCL-Gel 1. These results indicate that moderate Fe-TP deposition improved membrane mechanical strength without compromising flexibility, as all modified membranes retained elongation above 100%.

Surface wettability was then characterized by water contact angle (WCA) measurement (Fig. 3 G and H). A WCA of 90° is generally regarded as the boundary between hydrophobic and hydrophilic surfaces (Loubet et al., 2025). The pristine PLCL-Gel membrane showed a WCA of 115.3 ± 2.8°, indicating typical hydrophobicity. After Fe-TP modification, the WCA values decreased sharply to 44.0 ± 2.3°, 33.4 ± 1.4°, and 18.0 ± 1.1° for the Fe-TP/PLCL-Gel 1, 4, and 8 membranes, respectively. These values were all below 90°, demonstrating a clear transition from a hydrophobic to a hydrophilic surface.

### 3.4 Drug release capacity and environmental degradation of Fe-TP/PLCL-Gel electrospun membranes

The in vitro release profiles of the electrospun membranes are shown in Fig. 3 I. The results demonstrated a clear layer-dependent release behavior: with increasing assembly cycles, both the initial release rate and the cumulative release of TP increased markedly. In particular, the Fe-TP/PLCL-Gel 1 membrane released 3.63 ± 0.03 mg/mL TP at 1 h and 5.04 ± 0.21 mg/mL at 6 h, whereas the Fe-TP/PLCL-Gel 4 and Fe-TP/PLCL-Gel 8 membranes released 4.99 ± 0.04 mg/mL and 7.52 ± 0.02 mg/mL at 1 h, and 6.92 ± 0.11 mg/mL and 10.51 ± 0.18 mg/mL at 6 h, respectively. During the subsequent release period, the cumulative TP concentration continued to increase, reaching 7.22 ± 0.09 mg/mL, 8.66 ± 0.14 mg/mL, and 12.21 ± 0.04 mg/mL at 72 h for the Fe-TP/PLCL-Gel 1, Fe-TP/PLCL-Gel 4 and Fe-TP/PLCL-Gel 8 membranes, respectively. These results indicate that higher Fe-TP assembly cycles provided a larger TP reservoir and accelerated the early-stage release, resulting in a higher cumulative release over time.

Meanwhile, Fig. 3 J presents the degradation behavior of the membranes under soil burial conditions. The pristine PLCL-Gel membrane degraded the fastest, with the residual weight decreasing to 46.73 ± 0.17% on day 10 and complete degradation occurring by day 20. In contrast, all Fe-TP-loaded membranes exhibited slower degradation rates, and their residual weight generally increased with the number of assembly cycles. On day 10, the residual weights of the Fe-TP/PLCL-Gel 1, Fe-TP/PLCL-Gel 4 and Fe-TP/PLCL-Gel 8 membranes were 91.43 ± 0.82%, 92.39 ± 1.01%, and 95.09 ± 1.21%, respectively. By day 20, these groups still maintained residual weights of 86.22 ± 0.25%, 86.54 ± 1.14%, and 86.58 ± 1.17%, respectively, all of which were much higher than that of the pristine membrane. On day 30, the residual weight of the Fe-TP/PLCL-Gel 1 membrane decreased to 72.79 ± 1.09%, whereas the Fe-TP/PLCL-Gel 4 and Fe-TP/PLCL-Gel 8 membranes remained at 82.15 ± 0.65% and 83.14 ± 0.66%, respectively, demonstrating that higher assembly cycles further delayed material degradation. Overall, Fe-TP modification significantly improved the environmental stability of the electrospun membranes, and the tunable assembly cycles endowed the materials with controlled degradation characteristics, enabling them to maintain structural integrity during use while retaining the potential for natural degradation thereafter.

### 3.5 Antioxidant activity of Fe-TP/PLCL-Gel electrospun membranes

The antioxidant activity of the Fe-TP/PLCL-Gel membrane was evaluated using DPPH and hydroxyl radical scavenging assays. As shown in Fig. 4 A-B, the DPPH solution remained purple in the control and pristine PLCL-Gel groups, whereas the Fe-TP-modified groups showed obvious decolorization from purple to yellow, indicating efficient DPPH radical scavenging. Quantitatively, the DPPH scavenging rate of the pristine PLCL-Gel membrane was only 8.48 ± 11.82%, while those of the Fe-TP/PLCL-Gel 1, Fe-TP/PLCL-Gel 4 and Fe-TP/PLCL-Gel 8 membranes reached 80.89 ± 0.82%, 80.12 ± 0.19%, and 79.19 ± 0.17%, respectively. These values were all significantly higher than that of the pristine membrane, confirming that Fe-TP incorporation endowed the membranes with strong radical scavenging capability.

**Fig. 4:**
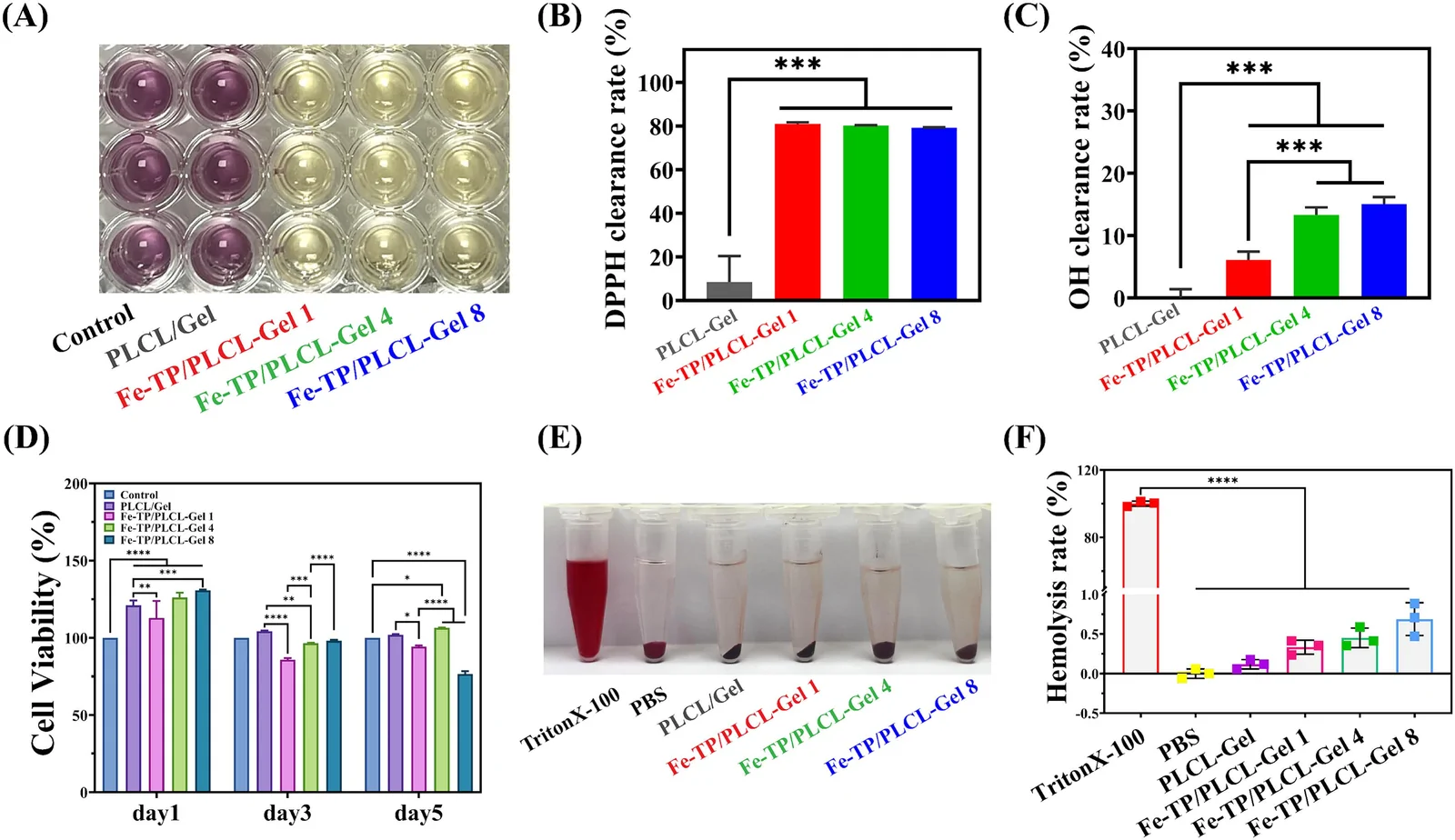
Antioxidant activity and preliminary biosafety of Fe-TP/PLCL-Gel electrospun membranes. (A) DPPH assay images; (B) DPPH radical scavenging activity; (C) Hydroxyl radical scavenging activity; (D) CCK-8 assay; (E) Hemolysis images; (F) Hemolysis rates. Data are mean ± SD (*n* = 3). *p < 0.05, **p < 0.01, ***p < 0.001, ****p < 0.0001.

The hydroxyl radical scavenging results (Fig. 4 C) showed a layer-dependent trend. The ·OH scavenging rates of the Fe-TP/PLCL-Gel 1, Fe-TP/PLCL-Gel 4, and Fe-TP/PLCL-Gel 8 membranes were 6.09 ± 1.35%, 13.34 ± 1.18%, and 15.04 ± 1.17%, respectively, indicating that higher Fe-TP loading enhanced hydroxyl radical scavenging. Taken together, Fe-TP modification significantly improved the antioxidant performance of the PLCL-Gel fibrous membranes, although the scavenging efficiency differed between radical systems.

### 3.6 Preliminary cytocompatibility and hemocompatibility of Fe-TP/PLCL-Gel electrospun membranes

Given that the fibrous membranes are intended for fruit preservation packaging, their safety under food-contact conditions is an important prerequisite for practical application. Therefore, the cytocompatibility of the membrane extracts was first evaluated using the CCK-8 assay (Fig. 4 D). The results showed that both the PLCL-Gel group and all Fe-TP-modified groups maintained relatively high cell viability at different culture time points, indicating that the extracts did not exert obvious inhibitory effects on cell proliferation. Among them, the Fe-TP/PLCL-Gel 4 sample continued to exhibit favorable cell proliferation-supporting ability at later stages of culture, whereas the cell viability of the Fe-TP/PLCL-Gel 8 sample decreased to 75.96% on day 3 but still remained above the commonly used cytotoxicity threshold, suggesting acceptable overall safety(Zhang et al., 2024). These results indicate that the Fe-TP-modified membranes possess good material safety and meet the basic requirements for subsequent fruit preservation applications.

In addition, a hemolysis assay was performed to further verify the contact safety of the membranes. As shown in Fig. 4 E, compared with the Triton X-100 positive control, the supernatants of the PBS group and all Fe-TP/PLCL-Gel groups remained relatively clear, and normal red blood cell sedimentation was observed, indicating that the membranes did not cause obvious hemolysis. Quantitative results (Fig. 4 F) further confirmed that the hemolysis rates of all samples were far below the 5% safety threshold, demonstrating good safety when the membranes were in contact with biological systems (Zhang, Sun, & Xu, 2025).Overall, among the samples with loading levels ranging from one to eight layers, the Fe-TP/PLCL-Gel fibrous membranes exhibited excellent food-contact safety and promising application potential.

### 3.7 Antibacterial activity of Fe-TP/PLCL-Gel electrospun membranes

Bacterial infection is an important factor affecting the postharvest preservation of fruits(Chavan et al., 2023). Therefore, the antibacterial performance of the Fe-TP/PLCL-Gel electrospun membranes was systematically evaluated by co-incubation plate counting, crystal violet biofilm staining, and live/dead fluorescence staining. The plate counting results are shown in Fig. 5 A and 5 B. The pristine PLCL-Gel membrane exhibited a certain inhibitory effect against both *S. aureus* and *E. coli*. However, with increasing Fe-TP assembly cycles, the antibacterial activity of the membranes was significantly enhanced. Quantitative analysis based on dilution-corrected CFU/mL values showed that the Fe-TP/PLCL-Gel 1 sample already exhibited a more pronounced antibacterial effect than the pristine membrane, while the Fe-TP/PLCL-Gel 4 and Fe-TP/PLCL-Gel 8 samples further improved the inhibition of both bacteria Among them, the Fe-TP/PLCL-Gel 8 sample showed the highest antibacterial efficiency, with the inhibitory effects against *S. aureus* and *E. coli* both approaching or exceeding 90% (Fig. 5 B). These results indicate that the incorporation of Fe-TP markedly improved the inhibitory effect of the membranes against the two representative bacterial strains, in a clear loading-cycle-dependent manner.

**Fig. 5:**
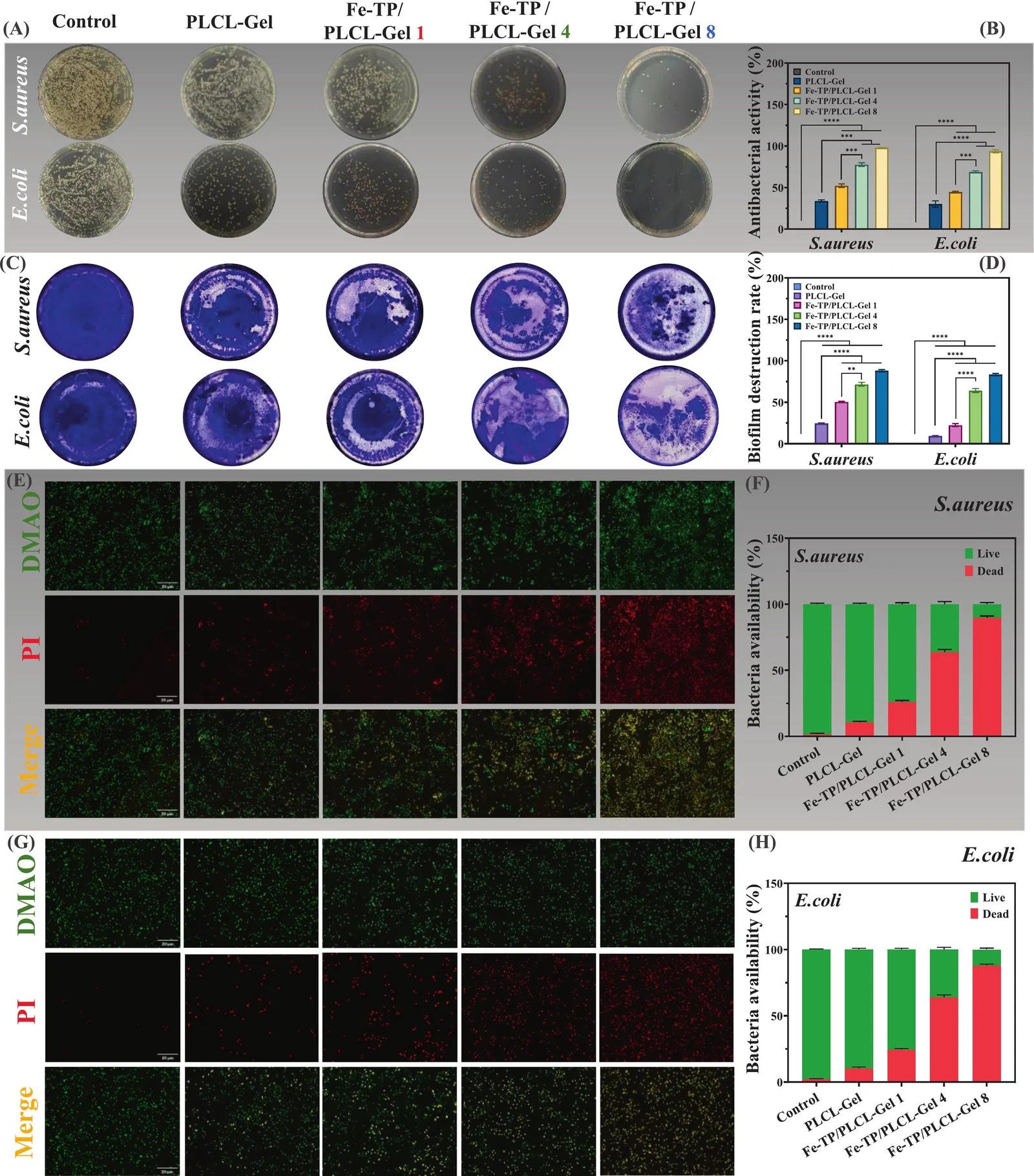
Antibacterial activity and biofilm inhibition of Fe-TP/PLCL-Gel electrospun membranes. (A) Plate-counting results, all groups for the same bacterial species were plated at the same dilution (10^−5^, 100 μL per plate); (B) Antibacterial efficiency calculated from dilution-corrected CFU/mL values; (C) Crystal violet staining of biofilms; (D) Biofilm biomass quantified at 570 nm; (E–H) Live/dead staining and quantification. Data are mean ± SD (*n* = 3). *p < 0.05, **p < 0.01, ***p < 0.001, ****p < 0.0001. (For interpretation of the references to color in this figure legend, the reader is referred to the web version of this article.)

The subsequent crystal violet staining results are shown in Fig. 5 C. All samples inhibited biofilm formation to different extents, and the inhibitory effect increased with the number of assembly cycles. Compared with the pristine membrane, the Fe-TP/PLCL-Gel 1 sample already significantly reduced the biofilm biomass, while the Fe-TP/PLCL-Gel 4 sample further enhanced biofilm inhibition against both *S. aureus* and *E. coli*, indicating a relatively favorable anti-biofilm capability. The biofilm scavenging rate of the Fe-TP/PLCL-Gel 4 sample increased to 71.52 ± 2.38% for *S. aureus* and 63.89 ± 2.54% for *E. coli*, whereas the Fe-TP/PLCL-Gel 8 sample exhibited the strongest biofilm inhibition (Fig. 5 D). The live/dead fluorescence staining results (Fig. 5 E–H) showed a similar layer-dependent trend, with a marked increase in the proportion of dead bacteria as the assembly cycles increased. In the Fe-TP/PLCL-Gel 4 sample, a large proportion of bacteria were already observed to be dead, while the Fe-TP/PLCL-Gel 8 sample rendered most bacteria nonviable, indicating a stronger antibacterial effect. These results suggest that Fe-TP modification not only inhibited planktonic bacterial growth, but also effectively weakened bacterial adhesion and biofilm formation on the membrane surface (Ribačuka et al., 2026).

Overall, Fe-TP modification significantly enhanced the antibacterial activity of the PLCL-Gel electrospun membranes against both Gram-positive and Gram-negative bacteria.

### 3.8 Evidence for the antibacterial action of Fe-TP/PLCL-Gel electrospun membranes

To obtain evidence related to the antibacterial action of the Fe-TP/PLCL-Gel membranes, bacterial morphology and intracellular component leakage were examined after treatment. As shown in Fig. 6 A, bacteria in the control group maintained typical intact morphologies, including short rod-shaped *E. coli* and spherical *S. aureus*, with smooth surfaces(Long et al., 2025). In contrast, both bacterial strains treated with Fe-TP/PLCL-Gel membranes displayed varying degrees of shrinkage, surface damage, and structural rupture, suggesting compromised cell-envelope integrity. The extent of morphological damage increased with the number of assembly cycles. Cell debris was observed after treatment with the Fe-TP/PLCL-Gel 1, while more pronounced structural collapse and fragmentation occurred in the Fe-TP/PLCL-Gel 4 and Fe-TP/PLCL-Gel 8 groups. *S. aureus* appeared more sensitive than *E. coli*, as almost no intact *S. aureus* cells were observed under the Fe-TP/PLCL-Gel 8 treatment, whereas a few residual *E. coli* structures remained.

**Fig. 6:**
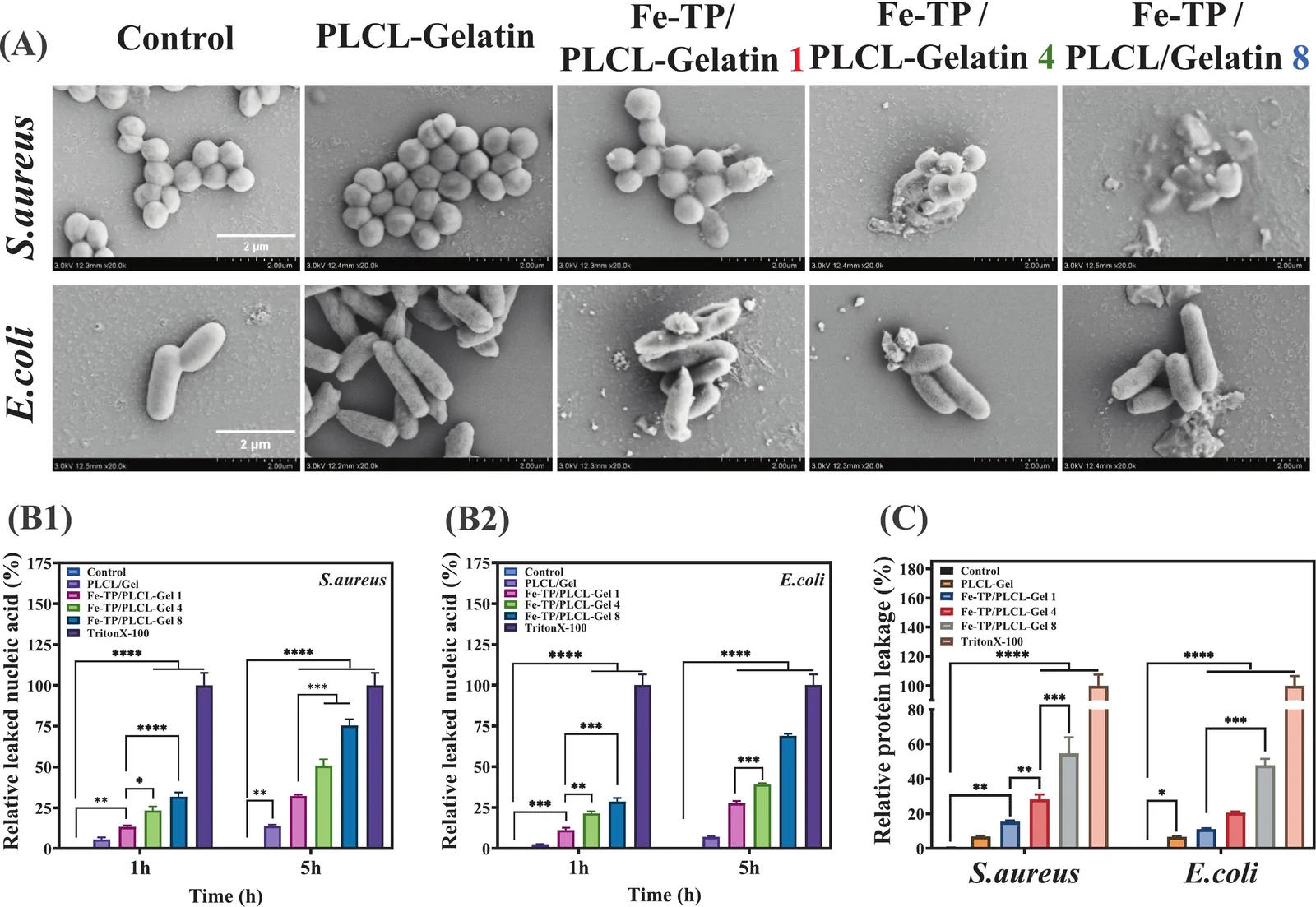
Antibacterial mechanism of Fe-TP/PLCL-Gel electrospun membranes. (A) SEM images after 2 h treatment; (B1, B2) Nucleic acid leakage at 1 and 5 h; (C) Protein leakage at 5 h. Data are mean ± SD (*n* = 5). *p < 0.05, **p < 0.01, ***p < 0.001, ****p < 0.0001.

In line with the SEM observations, the nucleic acid and protein leakage assays showed increased leakage consistent with compromised cell-envelope integrity. Relative nucleic acid leakage increased significantly with assembly cycles at both 1 and 5 h, and leakage levels at 5 h were generally higher than those at 1 h, which is consistent with the greater Fe-TP release from membranes with higher assembly cycles and the longer exposure time, leading to more severe damage to the bacterial membrane barrier. For *S. aureus* (Fig. 6 B1), the nucleic acid leakage values of the Fe-TP/PLCL-Gel 1, Fe-TP/PLCL-Gel 4, and Fe-TP/PLCL-Gel 8 membranes were 13.34 ± 0.75%, 23.45 ± 2.38%, and 31.77 ± 2.56% at 1 h, respectively, and increased to 32.19 ± 0.89%, 50.85 ± 3.90%, and 75.44 ± 3.89% at 5 h. *E. coli* showed a similar trend (Fig. 6 B2), with the Fe-TP/PLCL-Gel 8 membrane reaching nearly 70% nucleic acid leakage at 5 h. The protein leakage assay also followed a layer-dependent pattern, with the Fe-TP/PLCL-Gel 8 membrane inducing the highest protein release. The relative protein leakage of *S. aureus* and *E. coli* reached 54.67 ± 9.28% and 47.96 ± 3.63%, respectively (Fig. 6 C).

Because the CFU counting (4 h), SEM morphology (2 h), and leakage assays (1 and 5 h) were performed with different initial bacterial loads, exposure times, and endpoints, these datasets were compared for trend consistency rather than numerical agreement. Taken together, the morphological and leakage results were broadly consistent with the CFU-based antibacterial data, suggesting that cell-envelope damage and the resulting leakage of intracellular components may contribute to the antibacterial activity of Fe-TP/PLCL-Gel membranes. This effect became more pronounced as the number of Fe-TP assembly cycles increased, consistent with the greater Fe-TP release and stronger bactericidal activity observed for the Fe-TP/PLCL-Gel 4 and Fe-TP/PLCL-Gel 8 membranes.

### 3.9 Preservation performance of Fe-TP/PLCL-Gel electrospun membranes on yellow cherries

Yellow cherries were used as a model fruit to evaluate the practical preservation efficacy of the Fe-TP/PLCL-Gel electrospun membranes. The membranes were placed in direct contact with the cherry surfaces. Appearance changes, decay degree, and weight loss were monitored during storage. As shown in Fig. 7 A, each package contained six cherries, and three parallel groups were established for the commercial PE preservative film, the pristine PLCL-Gel membrane, and the Fe-TP/PLCL-Gel 1, Fe-TP/PLCL-Gel 4, and Fe-TP/PLCL-Gel 8 membranes.

**Fig. 7:**
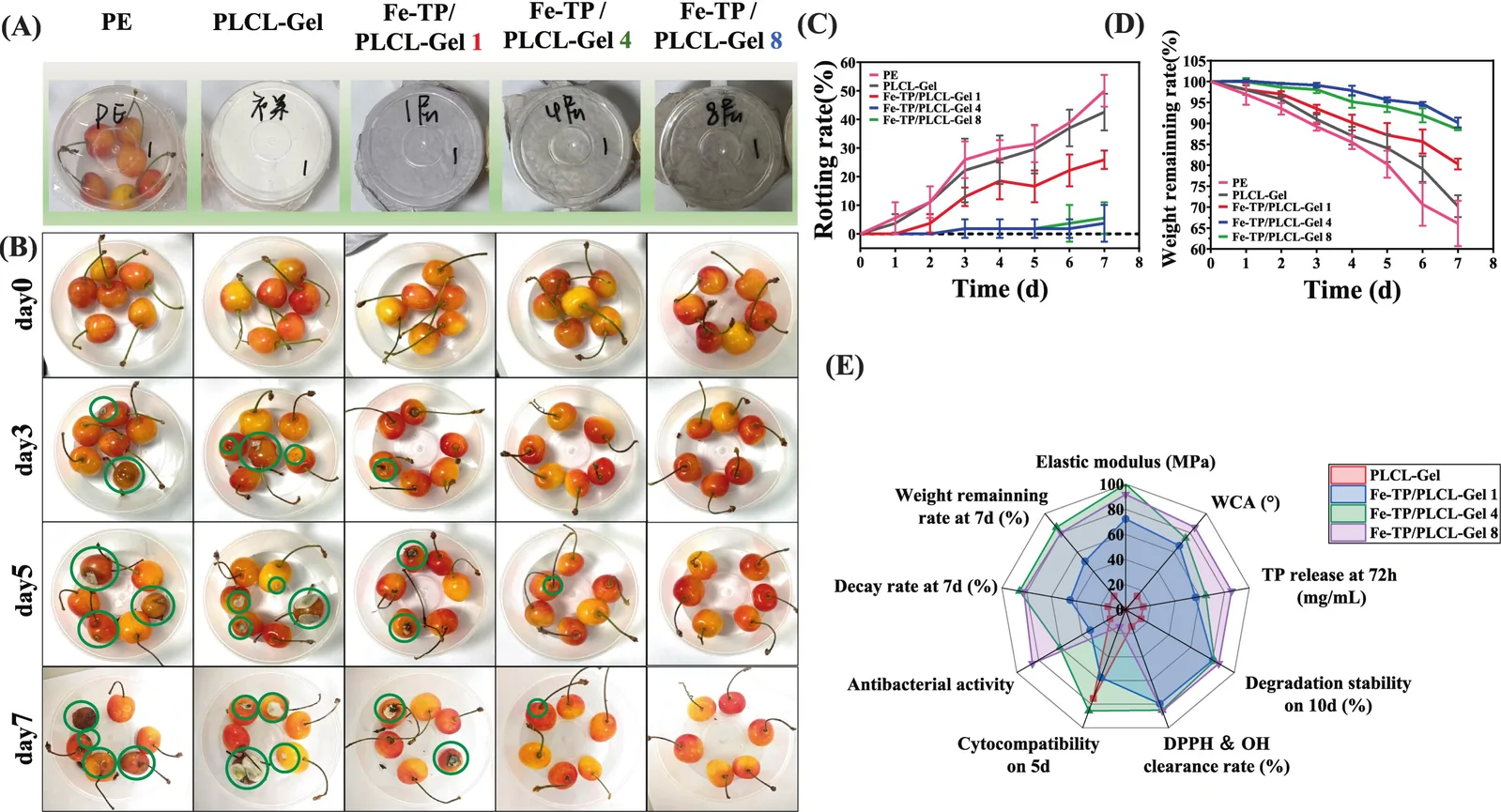
Preservation performance of Fe-TP/PLCL-Gel electrospun membranes on yellow sweet cherries. (A) Packaging schematic; (B) Representative images during storage; (C) Decay rate; (D) Weight remaining rate; (E) Radar plot of multi-indicator performance. All indicators were min–max normalized to 0–100 across the compared groups. Benefit-type indicators: higher values are preferable; cost-type indicators (water contact angle and decay rate at 7 d): lower values are preferable. All axes were displayed with equal visual weight, and no weighted composite score was calculated. Data are mean ± SD (*n* = 3). (For interpretation of the references to color in this figure legend, the reader is referred to the web version of this article.)

Representative images from day 0 to day 7 are presented in Fig. 7 B. The PE group and pristine PLCL-Gel group began to show visible decay on day 2. From day 3 to day 7, deterioration gradually spread to adjacent fruits and was accompanied by browning, softening, mold growth, odor changes, and juice leakage. In contrast, Fe-TP/PLCL-Gel membranes substantially delayed cherry decay. The Fe-TP/PLCL-Gel 4 and Fe-TP/PLCL-Gel 8 membranes exhibited the most pronounced preservation effects, maintaining relatively intact appearance and color after 7 days, with only slight localized mold spots. In particular, cherries packaged with the Fe-TP/PLCL-Gel 4 membrane showed only small mold spots near the peduncles of a few fruits, and the fruits maintained a relatively intact appearance and color.

The decay rate results were consistent with the visual observations (Fig. 7 C). The PE group showed a rapid increase in decay during the later storage stage, reaching 50.00 ± 5.56% on day 7. The pristine PLCL-Gel group also displayed progressive decay. In contrast, the Fe-TP/PLCL-Gel groups exhibited markedly lower decay rates. The Fe-TP/PLCL-Gel 4 membrane showed the best decay inhibition, with a decay rate of only 1.85 ± 3.21%, while the Fe-TP/PLCL-Gel 8 membrane reached 5.56 ± 5.56%, both significantly lower than the PE group. It should be noted that the decay rate of the Fe-TP/PLCL-Gel 8 membrane group began to increase after day 5, suggesting that although this membrane effectively delayed spoilage, some late-stage quality deterioration still occurred.

The weight remaining rate further reflected the changes in cherry quality during storage (Fig. 7 D). The PE and pristine membrane groups showed a continuous decline over time, reaching 66.11% and 70.21%, respectively, by day 7. In contrast, all Fe-TP-modified groups maintained higher weight remaining rates throughout the storage period. On day 7, the Fe-TP/PLCL-Gel 1, Fe-TP/PLCL-Gel 4, and Fe-TP/PLCL-Gel 8 samples retained 80.30%, 90.19%, and 88.60% of their initial weight, respectively. Notably, the Fe-TP/PLCL-Gel 4 consistently exhibited the highest weight remaining rate and the slowest decline during storage. Based on the min–max normalized indicators (Fig. 7 E), the Fe-TP/PLCL-Gel 4 membrane achieved the highest normalized scores in elastic modulus, cytocompatibility, decay rate, and weight remaining rate, with near-highest scores in degradation stability and antioxidant activity. Although the Fe-TP/PLCL-Gel 8 membrane showed stronger antibacterial activity and hydrophilicity, its cytocompatibility was markedly lower. Taken together, these results suggest that the Fe-TP/PLCL-Gel 4 membrane provided the most favorable overall balance among mechanical properties, release behavior, safety, and preservation efficacy, rather than maximizing any single indicator.

## 4 Discussion

To construct fruit packaging materials with both preservation activity and structural stability, Fe-TP/PLCL-Gel electrospun membranes were fabricated by combining electrospinning with layer-by-layer polyphenol–metal coordination assembly. SEM observations (Fig. 2 A) and fiber diameter analysis (Fig. 2 B), together with the appearance of Fe—O and phenolic C—O bands in the FTIR spectra (Fig. 2 D), confirmed the successful deposition of Fe-TP metal-phenolic networks on the membranes (Franco et al., 2021; Hu et al., 2025). With increasing loading cycles, the average fiber diameter increased, indicating gradual accumulation of the coating layer(Milisav et al., 2025). Cross-sectional SEM images (Fig. 2 C) further revealed that the Fe-TP coating was deposited primarily from the surface inward. The loaded layer became denser at higher assembly cycles without an obvious change in the overall membrane width, possibly indicating tighter complexation within the Fe-TP network. This trend is consistent with the fiber diameter analysis (Fig. 2 B), which showed a gradual increase in average diameter with increasing assembly cycles. XPS analysis (Fig. 2 E) confirmed the presence of Fe on the membrane surface and revealed a mixed Fe^2+^/Fe^3+^ state. The Fe^2+^ fraction increased from 44.95% for Fe-TP/PLCL-Gel 1 to 59.22% and 60.79% for Fe-TP/PLCL-Gel 4 and 8, respectively, indicating partial reduction of Fe^3+^ by tea polyphenols during assembly(Pan et al., 2022). These results support the formation of an Fe-TP metal–phenolic network rather than a pure Fe^3+^-TP complex. The Fe and TP loading contents, quantified by ICP-OES (Fig. 3 A) and the Folin–Ciocalteu method (Fig. 3 B), respectively, increased progressively with the number of assembly cycles, and the mass gain after Fe-TP loading followed the same trend (Fig. 3 C). These results demonstrate that the active loading could be tuned by controlling the assembly cycles. This structural modification was accompanied by improved wettability, likely due to the intrinsic hydrophilicity of tea polyphenol and the effective exposure of Fe-TP on the membrane surface (Fig. 3 G and H), consistent with our previous findings(Ye et al., 2025; Zhou et al., 2024). The multilayer coating strengthened the fibrous network, with the Fe-TP/PLCL-Gel 4 sample showing the highest elastic modulus (Fig. 3 E); the slightly lower modulus of the Fe-TP/PLCL-Gel 8 membrane may be associated with the small surface cracks and local heterogeneity observed by SEM, while all modified membranes still maintained elongation at break above 100% (Fig. 3 F) (Sharifzadeh et al., 2023). Overall, Fe-TP modification regulated membrane structure, surface wettability, and mechanical performance in a layer-dependent manner, while maintaining the robustness required for fruit preservation packaging(Al-Abduljabbar & Farooq, 2023; Olawade et al., 2024).

Fe-TP loading also regulated TP release and environmental stability in a layer-dependent manner. Higher assembly cycles led to a faster initial release and a larger cumulative release of TP (Fig. 3 I). This indicates that multilayer deposition increased the accessible TP reservoir and promoted stronger early-stage release while sustaining a higher total release over time(Quintero-Borregales et al., 2023; Wu et al., 2025). In soil burial tests (Fig. 3 J), Fe-TP modification significantly delayed mass loss, and higher assembly cycles generally increased the residual mass, which may be related to the denser MPN coating forming an effective barrier against the penetration of soil microorganisms and degradative enzymes(Ma et al., 2025; Pfohl et al., 2022).

The multilayer membranes exhibited pronounced antioxidant activity, as evidenced by consistently strong DPPH scavenging (Fig. 4 A and B) and a layer-dependent increase in ·OH scavenging capacity (Fig. 4 C). This behavior is likely attributable to the abundant phenolic hydroxyl groups in TP, which can donate hydrogen atoms or electrons to quench free radicals and may help delay oxidative browning and quality deterioration during fruit storage(Kumari et al., 2022; Wang, Duan, et al., 2024; Zhang et al., 2023). In addition, the CCK-8 (Fig. 4 D) and hemolysis assays (Fig. 4 E) confirmed that the Fe-TP-modified membranes maintained acceptable preliminary biosafety. Fe-TP modification markedly enhanced the antibacterial and anti-biofilm performance of the PLCL-Gel membranes in a loading-cycle-dependent manner. Plate counting (Fig. 5 A-B) showed that the inhibition efficiencies against both *S. aureus* and *E. coli* approached or exceeded 90% in the Fe-TP/PLCL-Gel 8 group, and crystal violet staining (Fig. 5 C and D) together with DMAO/PI imaging (Fig. 5 *E*-H) confirmed progressively weaker biofilm formation and a higher proportion of dead bacteria with increasing loading cycles. To obtain evidence related to the antibacterial action, SEM (Fig. 6 A) and intracellular leakage assays (Fig. 6 B and C) were further performed. The treated bacteria exhibited obvious morphological damage accompanied by increased leakage of nucleic acids and proteins, which is consistent with compromised cell-envelope integrity (Kadeřábková et al., 2023). However, these assays alone do not establish membrane disruption as the exclusive or primary bactericidal mechanism. In addition, the present study primarily evaluated antibacterial activity against *E. coli* and *S. aureus*. Further studies incorporating representative spoilage fungi and yeasts, as well as quantitative analysis of fungal and yeast populations on stored cherries, are needed to comprehensively evaluate the antimicrobial preservation performance of the developed membranes. Taken together, these results suggest that Fe-TP assembly can effectively improve antibacterial and anti-biofilm performance against representative Gram-positive and Gram-negative bacteria, while the preservation data suggest that moderate loading may provide a more favorable balance between antibacterial efficacy and practical fruit-preservation performance(Li, Yu, et al., 2025; Sun et al., 2025).

The yellow sweet cherry storage results further confirmed that preservation performance depends on a balance among antimicrobial activity, antioxidant capacity, release behavior, and interfacial regulation(Jama et al., 2026; Panou et al., 2025). From the perspective of yellow sweet cherry preservation, the in vitro functionality of the Fe-TP/PLCL-Gel membrane was further evaluated in a real fruit model. Compared with the commercial PE film and the pristine membrane, all Fe-TP/PLCL-Gel groups delayed decay, browning, and softening during storage, and the Fe-TP/PLCL-Gel 4 membrane achieved the most favorable overall preservation performance among the tested groups (Fig. 7 B). This indicates that fruit preservation depends on a balance among antibacterial activity, antioxidant capacity, TP release, and interfacial regulation rather than on antibacterial potency alone. Consistently, the Fe-TP/PLCL-Gel 4 sample showed the lowest decay rate (1.85 ± 3.21% at day 7) in Fig. 7 C, far below that of PE (50% ± 5.56%), while also maintaining the highest weight remaining rate (90.19%) in Fig. 7 D, followed by PE (66.11%). Although the Fe-TP/PLCL-Gel 8 membrane exhibited stronger antibacterial activity, its late-stage preservation stability was slightly inferior to that of the Fe-TP/PLCL-Gel 4 membrane. Notably, the Fe-TP/PLCL-Gel 4 membrane required only about half the active loading of the Fe-TP/PLCL-Gel 8 membrane, yet it already achieved favorable antibacterial, antioxidant, mechanical, and preservation performance, and even better late-stage preservation stability. This indicates that the Fe-TP/PLCL-Gel 4 membrane provides higher utilization efficiency of the active components and is therefore more economical for the tested application. Moreover, because no experimental evidence indicates that the Fe-TP/PLCL-Gel 8 membrane acts through a fundamentally different antibacterial mechanism from the Fe-TP/PLCL-Gel 4 membrane, the observed differences between them are more reasonably attributed to quantitative variations in active reservoir, release kinetics, hydrophilicity, and mechanical properties. Thus, Fe-TP/PLCL-Gel 4 offers the most favorable balance among antibacterial efficacy, preservation performance, and material utilization (D'Aquino et al., 2022; Song et al., 2025).

## 5 Conclusion

In this study, assembly-cycle-controlled Fe-TP metal-phenolic networks were successfully constructed on PLCL-Gel electrospun membranes, enabling tunable regulation of membrane wettability, release behavior, degradation stability, and fruit-preservation performance. Fe-TP modification markedly reduced the water contact angle from 115.3° to 33.43° and 18.01° for Fe-TP/PLCL-Gel 4 and Fe-TP/PLCL-Gel 8, respectively. Although Fe-TP/PLCL-Gel 8 exhibited stronger antibacterial activity (>90%) than the lower-layer samples, Fe-TP/PLCL-Gel 4 provided a more favorable balance of mechanical stability, hydrophilicity, sustained release, biosafety, and preservation efficacy. Notably, it reduced the decay rate of yellow cherries to 1.85% ± 3.21% after 7 days of storage, compared with approximately 50% for the PE control. These findings demonstrate that controlling the Fe-TP assembly cycle is important for balancing active release and fruit-preservation performance.

Nevertheless, several aspects require further investigation before practical application. Sensory quality and key physicochemical attributes of the preserved fruit should be systematically evaluated, while food-contact safety requires further validation through migration and toxicological studies. In addition, the antibacterial mechanism remains to be clarified using direct mechanistic evidence. Future studies should therefore focus on these aspects, together with performance under realistic cold-chain conditions and scalability, to further establish the practical potential of Fe-TP/PLCL-Gel membranes for active food packaging.

## CRediT authorship contribution statement

**Jiaqi Liu:** Writing – original draft. **Tingzi Yan:** Writing – review & editing. **Qiongyu Wang:** Data curation. **Jiaxuan Bao:** Visualization. **Yuchan Li:** Investigation. **Jigang Piao:** Methodology. **Hui Chai:** Project administration. **Guoying Zhou:** Resources.

## Declaration of competing interest

The authors declare that they have no known competing financial interests or personal relationships that could have appeared to influence the work reported in this paper.

## Data Availability

Data will be made available on request.
